# The 4^th^ National Neurology Forum | Interview with Prof. Paul Verschure

**DOI:** 10.25122/jml-2026-1010

**Published:** 2026-06

**Authors:** Stefana-Andrada Dobran, Alexandra Gherman

**Affiliations:** 1 RoNeuro Institute for Neurological Research and Diagnostic, Cluj-Napoca, Romania

**Interviewee: Prof. Paul Verschure**

**Interviewer: Ms. Stefana-Andrada Dobran**

## S.D.: Hello, Professor Paul Verschure, and welcome to the 4^th^ National Neurology Forum. First, please tell us a little bit about yourself

**P.V.:** I am really happy to be here. I am Paul Verschure, a distinguished university professor at Universidad Miguel Hernández in Elche, Spain. I started my career as a psychologist, then moved to Artificial Intelligence (AI) and robotics, and from there I transitioned to neuroscience. Now I am bringing all these fields together.

What brings me here is that, for the last twenty years, I have been on a mission to bring brain theory to the clinic. I am also interested in neurology, and particularly in stroke neurorehabilitation. Specifically, [I am interested] in how technology and Artificial Intelligence can be used to improve patient care and enhance recovery of stroke survivors.

## S.D.: What role does the National Neurology Forum play in advancing neurology and neurosciences?

**P.V.:** What is special about the federation here in Romania is that it is a very inclusive environment. It is very open, but it also tries to develop an agenda that is relevant to stroke care in Romania.

It is more than a professional organization or just a way for professionals to meet. Yes, it does that too. However, what I really respect and appreciate is that there is a real vision about generating impact and relevance, and that makes it a very special organization compared to others in Europe and the rest of the world.

## S.D.: You are the developer of the Rehabilitation Gaming System (RGS). How does Artificial Intelligence impact this field, in your perspective?

**P.V.:** Let's first look at what a rehabilitation gaming system is.

This started about 20 years ago, when we had this idea that we must look at the recovering brain from a *brain-centric perspective*. From the outside, you might see certain deficits. For example, a patient may have a motor deficit or a cognitive deficit, and we tend to categorize that [separately]. [We say] “This is a motor deficit; it is very different from a cognitive deficit.” However, if you look at it from the brain, there might be much more correlation and overlap between these deficits.

Our challenge then became “How do we deliver a more brain-centric intervention approach?” We started to think about basic principles of brain organization. For instance, the brain has goals. The brain interacts with the world in an embodied way. I wave my hands, for example – I'm not a disembodied intelligence. We brought around 20 of such principles together.

The next question was “How do we deliver this to a patient?” You can say “The intervention should be goal-oriented”, but how do I control these goals? Or you say, "I want the intervention to be personalized to the patient. Well, how do I do that?" Should the therapist always adjust things?

Then it became clear that the most effective technology to deliver that, the most effective way is through Virtual Reality and interaction. So, that is why RGS is all built around virtual reality scenarios. It is non-immersive, so it does not have head-mounted displays. If you look at the screen, you are projected into a virtual reality, and can perform tasks with a virtual body in a virtual environment.

One major advantage is that we control everything, so we can make sure that we provide the right [level of] stimulation, number of distractors, difficulty, and so on. This is how RGS started.

Of course, once patients begin interacting with the virtual reality system, performing tasks and recovering, a tremendous amount of data is generated. Then you can say “We want to also now analyze this data online as it comes in and immediately optimize the intervention”.

For example, you might see that the patient has a bad day. They have fatigue, for instance, so they have difficulties keeping up. Then you want an automated way to fine-tune this interaction. However, you cannot have a therapist always interfering. We just do not have the manpower for that.

This is where AI enters the picture. In my view, the future of neurorehabilitation is fundamentally digital. Patients will increasingly interact with virtual reality systems and algorithms that operate largely autonomously. These AI-driven systems will continuously optimize intervention protocols, training intensity, data analysis, and clinical reporting.

To achieve this, we need AI.

What I see happening is that AI, combined with other technologies such as Virtual Reality, will take over a lot of the individualized, difficult, mechanical tasks of rehabilitation and diagnostics. With AI today, we can give you higher quality and higher resolution diagnostics at home through the telephone than you would be able to generate in the clinic, because there is no time, there are not enough people. You also must transport the patient.

In this way, we can offload a significant amount of work from clinicians, neurologists, therapists, and other healthcare professionals to machines and AI. This allows them to focus on the tasks that are truly difficult: managing complex symptom combinations, maintaining the human relationship with the patient, and making nuanced clinical decisions.

So, what I see happening in the future is that neurology and neurological intervention will become more of a hybrid, collaborative environment where humans and AI algorithms work together with very clear roles in the process to deliver high quality, individualized care throughout the patient journey – from the first day of treatment to lifelong support.

## S.D.: What is next for RGS here in Romania?

**P.V.:** Working with our colleagues in Cluj and with other hospitals, we see an incredible opportunity to contribute to the Romanian healthcare system.

We know it is very challenging. There is never enough money, never enough specialists, and there are lots of patients. What I just sketched out is how machines, technology, and algorithms can offload the medical staff. That is something we are actively developing here in Romania, in a very serious way.

We have brought many stakeholders together, because this is a collaborative project. You cannot simply say “This is the best solution ever, now use it.” We need to involve clinicians, healthcare providers, reimbursers, and patient organizations to collectively build this new vision of brain health.

What I find amazing is the way we brought the stakeholders together – also thanks to the Federation of Neurology, and the network behind it. I believe this creates such an incredible opportunity to really make it work here in Romania and set an example for Europe and the rest of the world.

Yes, it is difficult. There are problems. There are limitations. But at least here, there is an interest in change, in finding a different way, because people are aware that we must overcome problems, and that is something you do not see everywhere.

I am very optimistic. I have to be optimistic because I always look into the future. But if I look at the interactions we are having, the discussions, the commitments we find among all the stakeholders, the leadership we have here in Romania, I think we have an incredible opportunity to do this together, to really, on the one hand, build a whole new quality of care in brain health and [on the other hand] set an example for Europe, which would be fantastic, and that is an opportunity we should just really embrace and just make happen.

## S.D.: Any further thoughts you would like to share with us?

**P.V.:** We are all trained to be specialists and people often are excellent specialists – good at what they do. But it is not enough, unfortunately.

If you look at a patient who has a stroke – first, there might be the ambulance personnel. There might be the General Practitioner (GP). You go to the hospital. You have acute care. You have neurosurgery. You have thrombolysis, thrombectomy, and so on. You have a very specific group of specialists to deal with.

Then you may be discharged or transferred to an inpatient care facility, where you interact with another set of specialists. After that, you return home and again interact with your GP and perhaps local support services.

All these specialists have different roles, and we are not sufficiently integrated. We need to develop a new culture of providing care – which is difficult, but it is a more inclusive, holistic, and integrative perspective – where we respect and understand each other's specialization and look at how we can help each other and be complementary, because one is not enough.

We have to do this together. We have to think outside of the box, and that is hard. To collaborate across these boundaries is hard, especially when you are under a lot of pressure. There is never enough time. You cannot stand still and think. It is important to find ways, maybe training programs, mentoring schemes, role models, whatever we can think of. We must build that culture in which we can work together. And what is so central in that is trust, leadership, but also having a common perspective.

I know people are worried about their own existence and their job, and they do not want to be stressed. But we must all try to jump out of that and say, "This is really a challenge we have to face together, so let us find a way to do it together."

Right now, we do not have good platforms for that, and I hope that also the Federation can really help us in achieving that next level of collaboration that we know we need, but we do not have the tools to do it yet.

For me, the technology story is, in some sense, just an operationalization and an implementation story on how we collaborate, because our technology goes along the whole patient journey, so it also means it is a way to connect all these specialists through that pipeline, and I think that is another important element that we have to work towards.

